# Autophagy and Age-Related Eye Diseases

**DOI:** 10.1155/2019/5763658

**Published:** 2019-12-14

**Authors:** Xue Yang, Xinan Pan, Xiaorui Zhao, Jin Luo, Mingpu Xu, Daoming Bai, Yan Hu, Xu Liu, Qiongfang Yu, Dian Gao

**Affiliations:** ^1^Department of Pathogen Biology and Immunology, Medical College of Nanchang University, Nanchang 330006, China; ^2^Second Clinical Medical College of Nanchang University, Nanchang 330006, China; ^3^Department of Gastroenterology and Hepatology, Second Affiliated Hospital of Nanchang University, Nanchang 330006, China

## Abstract

**Background:**

Autophagy is a catabolic process that depends on the lysosome. It is usually used to maintain cellular homeostasis, survival and development by degrading abnormal substances and dysfunctional organelles, especially when the cell is exposed to starvation or other stresses. Increasing studies have reported that autophagy is associated with various eye diseases, of which aging is one of the important factors.

**Objective:**

To summarize the functional and regulatory role of autophagy in ocular diseases with aging, and discuss the possibility of autophagy-targeted therapy in age-related diseases.

**Methods:**

PubMed searches were performed to identify relevant articles published mostly in the last 5 years. The key words were used to retrieve including “autophagy”, “aging”, “oxidative stress AND autophagy”, “dry eye AND autophagy”, “corneal disease AND autophagy”, “glaucoma AND autophagy”, “cataract AND autophagy”, “AMD AND autophagy”, “cardiovascular diseases AND autophagy”, “diabetes AND autophagy”. After being classified and assessed, the most relevant full texts in English were chosen.

**Results:**

Apart from review articles, more than two research articles for each age-related eye diseases related to autophagy were retrieved. We only included the most relevant and recent studies for summary and discussion.

**Conclusion:**

Autophagy has both protective and detrimental effects on the progress of age-related eye diseases. Different types of studies based on certain situations in vitro showed distinct results, which do not necessarily coincide with the actual situation in human bodies completely. It means the exact role and regulatory function of autophagy in ocular diseases remains largely unknown. Although autophagy as a potential therapeutic target has been proposed, many problems still need to be solved before it applies to clinical practice.

## 1. Introduction

Autophagy, an evolutionarily conserved catabolic process responsible for lysosome-dependent degradation and recycling, plays an important role in maintaining intracellular homeostasis and viability in eukaryotic organisms [1]. During the cellular autophagy process, some damaged cytoplasmic proteins and organelles can be degraded to meet the metabolic needs of the cell and renew some organelles. Thus, autophagy can be induced by diverse stress signals, such as starvation, hypoxia, ionizing radiation, chemotherapeutic agents, and infection [2]. Sometimes, autophagy can function as a protective mechanism in cells. However, excessive self-degradation can be deleterious and lead to autophagic cell death, which is called type 2 programmed death. Previous studies have revealed that some signaling pathways can function as a switch in regulating cell survival or cell death in autophagy [3]. Therefore, a growing number of studies have explored the relationship between autophagy and human diseases. The mechanism of defective dead cell clearance in autophagy is associated with inflammatory diseases [4], neurodegenerative disease [5], etc. In most cases, autophagy may play positive and negative roles, e.g., in cancer and a variety of age-related eye diseases. In this comprehensive review, we focus on the role of autophagy in the age-associated pathological changes of eye diseases, including cornea, lens, and retina.

## 2. Methods

The PubMed database was used for the literature search in this review. The search strategy was limited to articles in English and used the following keywords: “autophagy”, “aging”, “oxidative stress AND autophagy”, “dry eye and autophagy”, “corneal disease AND autophagy”, “glaucoma AND autophagy”, “cataract AND autophagy”, “AMD AND autophagy”, “cardiovascular diseases AND autophagy”, “diabetes AND autophagy”. Other related terms and references were searched if necessary. Filters were set to retrieve articles published mostly in the last 5 years which are available in full text. The articles were classified, assessed, selected and then excluded articles about “autophagy in nonage-related eye diseases”.

## 3. Results

Among the most relevant articles of autophagy and age-related eye diseases, recent studies were discussed as follows in our review primarily (Supplementary [Supplementary-material supplementary-material-1]).

### 3.1. Autophagy

#### 3.1.1. The Types of Autophagy

**Figure 1:**
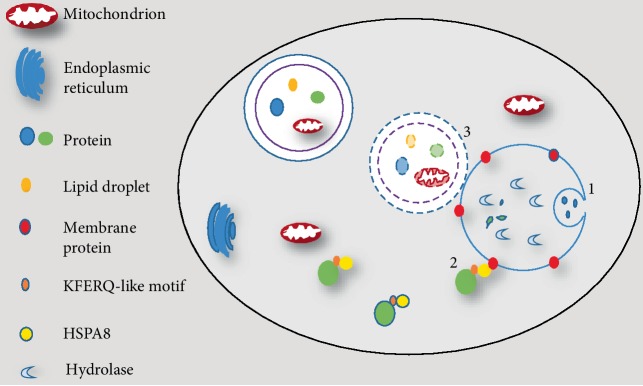
Three types of autophagy. (1) In microautophagy, proteins or other targeted components are translocated into the lysosomes for degradation via direct invagination of the lysosomal limiting membrane. (2) In chaperone-mediated autophagy, substrate proteins carrying a KFERQ-like pentapeptide sequence are firstly recognized by the Hsc70 chaperones, which then binds with integral lysosome membrane protein LAMP-2A. The complex is finally translocated into lysosomal lumen. (3) In macroautophagy, proteins, dysfunctional organelles, and random cytoplasm are enclosed is enclosed phagophore to form an autophagosome, which then fuses with lysosomes to complete the degradation of cargoes.

Autophagy is a cellular process in which cytoplasmic contents are degraded within the lysosome/vacuole, and then macromolecular constituents are recycled [6]. There are three common forms of autophagy: microautophagy, chaperone-mediated autophagy (CMA), and macroautophagy. They differ in the way how the cargo is transferred to lysosomes. For microautophagy, cytosolic material is translocated into the lysosomes for degradation by direct invagination or septation of the lysosomal limiting membrane [7]. CMA is a process in which cytosolic proteins binding to molecular chaperones are transported to the lysosomal lumen for digestion. CMA degrades only soluble proteins bearing a KFERQ-like motif bound to constitutive member of hsp70 chaperone family but does not affect organelles or other macromolecules [8]. Macroautophagy, a typical representative of autophagy (Figure 1), plays a crucial role in the process of human physiology and pathology, including development, aging, programmed cell death, and some diseases. Autophagy can be further divided into two categories: nonselective autophagy and selective autophagy [9]. Nonselective autophagy is primarily regarded as a survival mechanism under starvation response. It serves to recycle fundamental building blocks to compensate for the lack of nutrients, whereas selective autophagy is specifically involved in eliminating damaged an organelle, a protein complex or other harmful substances [10]. Micro- and macroautophagic responses can degrade cytoplasmic components in a relatively nonselective manner [1]. Additionally, all microautophagy, macroautophagy, and CMA can also operate in a selective manner through a mechanism involving the recognition of autophagy substrates by dedicated receptors [11]. For example, various types of cargoes are selectively degraded through macroautophagy, including peroxisomes (pexophagy), ribosomes (ribophagy), protein aggregates (aggrephagy), pathogens (xenophagy), and mitochondria (mitophagy) [12]. Among these cargo-specific types of autophagy, mitophagy is one of the best described type in part, because dysfunction of mitophagy have been connected to the development of several pathologies, including neurodegenerative disorders and some age-related eye diseases.

#### 3.1.2. The Process and Regulation of Autophagy

The process and molecular mechanisms of microautophagy, CMA and macroautophagy are different. Here, we mainly introduce macroautophagy. Compared with microautophagy and CMA, the most critical difference in macroautophagy (hereafter referred to as “autophagy”) is the sequestration of cytoplasmic cargoes by a new compartment, the autophagosome [13]. Currently, more than 30 autophagy-related (ATG) proteins have been identified in yeast and mammals [14]. Some of them have been identified as the core machinery for the formation of autophagosomes. These core ATG proteins are composed of five general protein complexes: (1) The ATG1/unc-51-like kinase 1 (ULK1) complex; (2) the ATG12 conjugation system; (3) the ATG8/LC3 conjugation/deconjugation system; (4) the phosphatidylinositol 3-kinase (PI3K) complex; and (5) two transmembrane proteins, ATG9/mATG9 and VMP1 [15]. These complexes play a central role in autophagosome formation.

**Figure 2:**
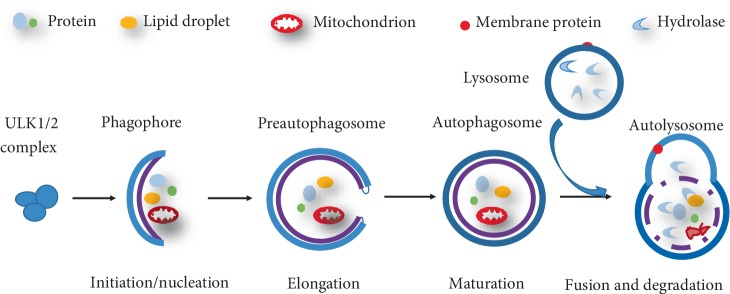
The classical process of autophagy. (1) Initiation/nucleation: autophagy induced by nutrient starvation initiates isolation membrane formation to engulf proteins and damaged organelles in the cytoplasm. This process is referred to as phagophore formation. (2) Elongation and maturation: the phagophore gradually elongates into a mature, closed autophagosome. (3) Fusion and degradation: the autophagosome fuses with a lysosome to form an autolysosome, and then cargoes are degraded by lysosomal enzymes such as proteases and lipases.

Generally, the classical process of autophagy can be summarized in five steps: initiation/nucleation, elongation, maturation, fusion, and degradation (Figure 2). We take mammalian autophagy as an example to briefly illustrate its core molecular mechanism and signal regulation. First, various cellular stresses can induce autophagy and cell starvation is common situation. Under starvation conditions, ATP levels are reduced, and consequently, AMP levels are increased. With the reduced ratio of ATP/AMP, AMPK is activated and then induces phosphorylation events on ULK1 [16]. The ULK1 complex, composed of ATG13, family interacting protein of 200 kDa (FIP200) and ATG101, is a key upstream regulatory factor in the initiation steps of autophagy [17]. ULK1 assembles at ATG9-containing membranes, which is followed by ULK1-dependent ATG9 phosphorylation [18]. Subsequently, the nucleation of vesicle is initiated, upon incorporation of phospholipids, which allows for the recruitment of a multiprotein complex with PI3K activity [1]. Vacuolar protein sorting 34 (VPS34) is a part of Class III PI3K, which synthesizes phosphatidylinositol 3-phosphate (PI3P) to support the process of autophagosomal elongation [19]. Finally, autophagosomal membranes are completely closed by engaging PI3P-binding ATG proteins and members of the WD-repeat protein interacting with phosphoinositides (WIPI) family [20]. Actually, the elongation and closure of the preauphagosome are more complicated than above molecular mechanism. Two ubiquitin-like protein conjugation pathways, ATG12 and LC3 conjugation system mediate the processes. LC3 is cleaved at its C terminus by Atg4 and becomes the cytosolic LC3-I. Then LC3-I is conjugated with phosphatidylethanolamine (PE) to become LC3-II, attached to both faces of the autophagosome by a ubiquitination-like enzymatic reaction. Therefore, LC3-II/LC3-I is usually used as a marker for assessing autophagic activity [15, 21, 22]. The matured autophagosome can move to and fuses with lysosome, which then forms autolysosome. The autophagic substrates are eventually degraded by lysosomal hydrolases for energy production. Together with LC3, lysosomal marker lysosomal-associated membrane protein 1 (LAMP-1) has also been used to assess autophagy [23]. It is also worth mentioning that the mammalian target of rapamycin (mTOR), a serine/threonine kinase, is able to suppress autophagosome formation by catalyzing the inactivating phosphorylation of ULK1 [24].

### 3.2. Age-Related Eye Diseases

**Figure 3:**
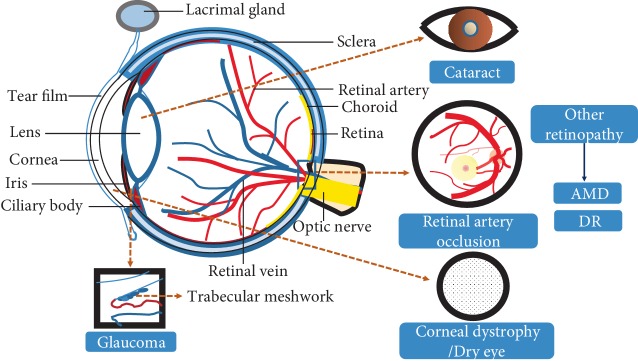
The eye structures. The main structure of eyes includes the cornea, lens, vitreous body, and retina. The cornea is related to some ocular surface diseases, such as dry eye and corneal dystrophy. A decline in lens transparency can lead to cataracts. Many eye diseases are related to injury of the retina, such as AMD, DR, RA, etc.

The delicate and intricate structure of the eyeball leads to the complexity and diversity of eye diseases (Figure 3). It has been reported that visual impairment is one of the leading disorders in older adults; it affects 246 million people worldwide and leads to an additional 39 million cases of blindness. Globally, the major causes of visual impairment are refractive errors (43%), cataract (33%), and glaucoma (2%). Sixty-five percent of all people suffering from visual impairment are aged 50 and older [25]. Therefore, aging is a major risk factor for a variety of ocular disorders, including age-related macular degeneration in the retina, cataracts in the lens, glaucoma in the optic nerve, dry eye syndrome in the cornea, and corneal dystrophy [26]. With aging, a series of changes occur in the diverse tissues of the eye, including dysregulated mitochondrial metabolism, shifted patterns in the utilization of glucose and lipids, accumulated methylated metabolites, and impaired taurine metabolism. All these changes may be attributed to the occurrence of eye diseases [26].

### 3.3. Oxidative Stress and Autophagy in Aging and Age-Related Eye Diseases

Several hypotheses have been proposed to explain the aging process, including increased oxidative stress, decreased mitochondrial energy metabolism, and accumulated mutations. Among them, the oxidative stress theory remains the most popular hypothesis of aging [27]. With aging, the concentration of endogenous antioxidant (AOX) sources is significantly decreased, while the production of reactive oxygen species (ROS) is increased [28]. The overproduction of ROS causes structural damage to biomolecules including DNA and protein, and hinders the repair process of damaged nuclear and mitochondrial DNA, which contributes to cell genomic instability, cell senescence, and the occurrence of age-related diseases [29]. Gradually, chronic cellular damage leads to a lot of cargoes and diminished autophagic flux in cells. Therefore, autophagy is insufficient with aging. The imbalance of redox is regarded as the common pathogenic mechanism in several age-related eye diseases, such as ocular surface diseases, glaucoma, age-related macular degeneration, and retinopathies.

Some evidence indicates that ROS can induce autophagy via mTOR-dependent pathway [30, 31]. The primary source of ROS as autophagy inducer originates from the mitochondrial respiratory chain. However, excessive ROS results in mitophagy by triggering mitochondrial oxidative stress [32, 33]. Oxidative stress and autophagy serve as protective or detrimental roles in response to cellular stressors depending on specific situation. In common, moderate changes in ROS restore cellular homeostasis and remove oxidative damage by inducing autophagy, which achieves protective effects, whereas acute and persistent ROS production restrains the function of lysosome, which causes accumulation of oxidative macromolecules in cells [34]. It also activates DNA damage response and is concomitantly signaled to the autophagy for orchestrating the response. However, whether autophagy delay or accelerate cell death is still unclear in this process [32]. It is worth noting that oxidative stress is just one of factors related to autophagy in age-related eye diseases.

#### 3.3.1. Dry Eye Disease

Dry eye disease, a multifactorial disease of tear film disorder, is caused by the loss of tears induced by deficient aqueous production and over evaporation. The typical clinical symptoms include irritation, redness, discharge, and easily fatigued eyes. Dry eye disease affects 5–34% of the global population [35], and its prevalence is increased significantly with age, especially in people over 65 years of age [36].

Evidence shows that constant exposure to oxidative stress is involved in dry eye disease [37]. Oxidative stress in corneal epithelial cells can reduce oxidative damage by inducing autophagy. Besides imbalance between oxidant and AOX sources, chronic inflammation with aging also contributes to dry eye [38]. Inflammatory factor interleukin-1 family members play a dominant role in damaging lacrimal gland functions [39]. In the process of inflammation injury, autophagy engages in the loss of lacrimal gland acinar cells in the early stage of inflammation. In an inflammation model established by injecting recombinant human interleukin-1*α* (IL-1*α*) into the exorbital lacrimal glands of female BALB/c mice [23], autophagic vacuoles, rather than secretory granules, were observed in the cytoplasm of lacrimal gland acinar cells from IL-1*α*—injected mice compared to the cells from saline-injected mice. In addition, LAMP-1 and microtubule-associated protein-1 light chain 3 (LC3) were both increased in the same cell. These results show that the autophagy and the lysosomal compartment involve in inflammatory-mediated dry eye disease [23]. In another study, impression cytology (IC) was used to detect the ratio of LC3 between the nucleus and cytoplasm. The result showed that the ratio was lower in aqueous tear-deficient dry eye cells than in healthy eyes, which further demonstrates a higher degree of autophagic cellular stress in aqueous tear-deficient dry eye cells [40].

Some studies have reported that proteins associated with ocular surface diseases also have effects on autophagy. Lacritin, a multifunctional tear glycoprotein [41], can increase the secretion of tears. It triggers autophagy in cultured corneal epithelial cells to restore cellular homeostasis in response to inflammatory cytokines interferon-*γ* and tumor necrosis factor, which are both sources of stress in dry eye. In this process, autophagic flux is initiated by lacritin-stimulated acetylation of forkhead box O3 (FOXO3) as a novel ATG101 ligand. Meanwhile, kinetically slower lacritin-dependent coupling of stress-acetylated FOXO1 to ATG7 is involved in the process of autophagy [42]. However, some studies have shown that lacritin monomers are selectively deficient in human dry eye [43], which leads to autophagic dysfunction in corneal epithelial cells during inflammation. Based on its function, the use of exogenous lacritin to stimulate autophagy could be a potential treatment approach for dry eye. Generally, autophagy plays a positive role in maintaining the health of the ocular surface. Under inflammatory conditions, autophagy can eliminate damaged acinar cells to control homeostasis of the lacrimal gland and protect corneal epithelial cells from inflammation-mediated damage. The impairment of autophagy may lead to the occurrence and aggravation of dry eye.

#### 3.3.2. Corneal Dystrophy

*Type 2 Granular Corneal Dystrophy*. Type 2 granular corneal dystrophy (GCD2), is a corneal stromal dystrophy subject to autosomal dominant inheritance. It is caused by the point mutation R124H in the transforming growth factor *β*-induced gene (TGF*β*1) [44]. Generally, TGF*β*1 is degraded by a basal level of autophagy in corneal fibroblasts to maintain corneal transparency [45–47]. In GCD2 patients, autophagy is activated in corneal fibroblasts, which show degenerative features, but mutant TGF*β*1 accumulates heavily in autophagosomes and/or lysosomes [48, 49]. Rapamycin, an autophagy activator, can reduce mutant-TGF*β*1 accumulation by increasing autophagic flux [48]. Further studies have revealed that the elevation of LC3-II and autophagosome levels could be due to delayed autophagic flux. This means that fusion between autophagosomes and lysosomes is impaired, and the deficient autophagy–lysosome pathway is attributed to the intracellular accumulation of mutant-TGF*β*1 during the pathogenesis of GCD2 [48]. The role and potential therapeutic benefits of autophagy in GCD2 should be considered in the future.

*Fuchs Endothelial Corneal Dystrophy*. FECD is the most common age-related progressive loss of human corneal endothelial cells (CECs) [50]. It usually occurs at the age of 40–50 years [51]. Genetic studies show that there are multiple gene mutations related to FECD. Missense mutations Q455K and L450W in the gene encoding the *α*2 subunit of collagen VIII (COL8A2) are associated with an early-onset form of FECD. COL8A2 is the primary component of the Descemet membrane (DM) [52, 53]. In the corneas of FECD patients, DM thickening is caused by abnormal COL8A2 aggregation [54]. DNA damage-regulated autophagy modulator 1 (Dram1) and transmembrane protein 74 (Tmem74) contribute crucial roles to autophagy activation [55, 56]. The expression levels of both genes are increased in the endothelia of COL8A2 L450W knock-in homozygous and COL8A2 Q455K knock-in homozygous mouse models. These results suggest a role for altered autophagy in this disease [57]. The function of autophagy is to reduce oxidative stress in FECD [58, 59]. Lithium has been proposed as a treatment for neurodegenerative diseases by inducing autophagy [60]. In a study, the CEC density of COL8A2 Q455K mice treated with lithium was higher than that of untreated mice; the mRNA transcript levels of P62 (an autophagy marker) and Tmem74 and the protein levels of the Atg5–Atg12 conjugate were upregulated. These results indicate that lithium may contribute to CEC survival by inducing autophagy and provide evidence of a potential medical treatment for FECD [59]. However, whether lithium can improve FECD caused by other genetic mutations is still unknown. Further studies should be carried out to find a therapy for FECD.

Moreover, mitophagy, as a type of selective autophagy, participates in the pathological changes of FECD [61]. A study found that CECs from FECD specimens exhibited extensive mitochondrial and nuclear DNA damage and increased autophagy levels. Furthermore, the result of transmission electron microscopy (TEM) revealed autophagosomes containing mitochondria, which showed that mitochondrial DNA damage induced mitophagy [62]. The increased mitophagy triggered the degradation of mitochondrial fusion protein mitofusin 2 (Mfn2), which led to more mitochondrial fragments [62]. Mitophagy was further induced by the increased number of mitochondrial fragments to clear these damaged mitochondria. All these processes resulted in a reduction in mitochondria in FECD compared with normal endothelial cells [62].

#### 3.3.3. Glaucoma

Glaucoma is the second leading cause of irreversible blindness in the world and age is an established risk factor [63, 64]. It is an optic neuropathy involving in the progressive degeneration of retinal ganglion cells (RGCs). Oxidative stress serves as a crucial role in glaucoma development initially by injuring TM cells which modulate the exit of aqueous humor (AH) from the anterior chamber of the eye and are helpful for maintaining intraocular pressure (IOP) [65]. In the natural course of aging, the increase of ROS can decrease the number of trabecular-meshwork (TM) cells [66]. Furthermore, oxidative damage to TM with aging also results in the resistance to aqueous humor outflow, which causes an elevation in intraocular pressure (IOP) [67]. The increased IOP compresses optic nerve axonal on the lamina cribrosa. It also obstructs axoplasmic flow and disturbs the process of retrograde neurotrophin transport to retinal ganglion cells (RGCs). These results contribute to apoptotic cell death, progressive degeneration of retinal tissues and development of glaucoma [68]. Persistent high IOP can promote activation of regulatory pathways in TM cells. For example, it can induce mTOR-independent autophagy, which is possibly helpful to maintain TM cellular homeostasis and cope with mechanical forces to TM [69]. However, oxidative stress caused by chronic elevation of IOP in glaucoma exerts an opposing influence on autophagy. In a chronic ocular hypertension model, cellular lipofuscin was elevated, whereas steady-state levels of LC3-II and proteolysis of long-lived proteins were reduced in TM cells isolated from glaucomatous cultures. Furthermore, the glaucomatous cultures failed to activate autophagy when exposed to hyperoxic conditions. The reduced autophagic capacity was related to the mTOR-dependent pathway [70]. Dysregulated autophagy can harm the outflow path of TM and promote deterioration of glaucoma.

In some cases of glaucoma, IOP is within the normal range; this is termed normal tension glaucoma (NTG) and is a subset of primary open angle glaucoma (POAG). Several gene mutations are linked to NTG, among them is optineurin (OPTN), which can mediate cargo-selective and nonselective autophagy as an autophagy receptor [71–74]. OPTN loci mutations include E50K and M98K [75]. A previous study showed that the expression of LC3-II in mutant M98K-expressing cells is increased. The elevation in autophagy flux results in RGC-5 death [76]. The transgenic expression of the E50K mutant in mice leads to elevated LC3-II levels and loss of RGCs with aging. Moreover, decreased mitochondria and increased autophagosome formation are detected in axons of the glial lamina, implying that death of these cells is associated with mitophagy [77]. The two mutations exhibit some difference in RGC death: E50K-OPTN induces RGC death by blocking autophagy flux, whereas M98K-OPTN induces autophagy-dependent retinal cell death [76, 78]. Understanding the exact roles of autophagy in different situations may help to identify novel glaucoma treatments.

#### 3.3.4. Cataract

Age-related cataracts, the most common type of cataract, usually occur in middle-aged and elderly people over 50 years of age. Cataracts form when lens transparency decreases. The eye lens constitutes a layer of epithelial cells (LECs) that overlay a series of differentiating fiber cells. Lens transparency depends on the metabolic activity of mitochondria in the lens epithelial cells and in the immature fiber cells [79]. Autophagic vesicles containing multilamellar membranes and mitochondrial fragments have been observed in LECs and differentiating fiber cells, which shows that autophagy and mitophagy contribute to maintaining lens homeostasis and transparency [79]. Therefore, it is possible that failure to eliminate increased numbers of mitochondrial fragments by mitophagy in LECs increases ROS and disrupts lens homeostasis. Gradually, lens transparency declines, and cataracts are formed.

However, the overactive autophagic response in the lens under oxidative stress leads to a loss of cell viability, which further promotes cataract development [80]. Thioredoxin binding protein-2 (TBP-2), a 46 kDa protein, is ubiquitously expressed primarily in the cytosol of many tissues, such as lens [81]. Thioredoxin (Trx), a 12 kDa protein that is present in all living cells, can reduce various substances to maintain the intracellular redox balance. TBP-2, a negative regulator of Trx, is overexpressed in cataracts. TBP-2 decreases the effect of Trx and increases oxidative stress in lens [82]. Overexpression of TBP-2 can increase oxidative stress, which results in an excessive autophagic response, followed by LEC death [80]. Furthermore, TBP-2 inhibits Akt/Bcl-xL signaling, independent of mTOR, to facilitate the initial stage of autophagy [80]. These findings will inspire more studies to explore target therapies for preventing autophagy induced by ROS in LECs and prevent cataract formation in elderly people [83].

#### 3.3.5. Retinal Diseases

*Age-Related Macular Degeneration (AMD)*. Age-related macular degeneration (AMD) is the major reason for blindness among elderly people in developed countries. It can be divided into two types: dry and wet. The dry form with drusen in the macula without choroidal neovascular formation is more common, while wet form is characterized by abnormal vascular endothelial growth factor (VEGF) secretion and neovascularization of macula [84]. The most important hallmark of both forms is degeneration of macular retinal pigment epithelial (RPE) cells.

AMD is a multifactorial progressive disease that results from both genetic and environmental insults. Chronic inflammation and oxidative stress are involved in it, although the exact mechanism of its pathogenesis is not fully clear [85–88]. Because high metabolic activity, abundant consumption of oxygen and high proportion of polyunsaturated fatty acids in RPE, RPE is more sensitive to oxidative stress damage [89]. Oxidative-induced autophagy can be observed in RPE in vitro. The up-regulated expression of KRT8 (keratin 8, an epithelial marker protein) is accompanied with autophagy under oxidative stress. During this process, KRT8 promotes autophagosome-lysosome fusion [90]. In addition, autophagy can be also stimulated by oxidative stress-induced p62 expression in RPE cells [91]. All these changes did not cause cell death, and autophagy contributes to a cytoprotective role. However, in some conditions, autophagy induced by oxidative stress can play an opposite role in RPE cells. For example, H_2_O_2_ treatment triggers autophagy in ARPE-19 cells, which inhibits cell proliferation [92, 93]. Erb-b2 receptor tyrosine-protein kinase 2 (ERBB2), a tyrosine kinase, belongs to the epidermal growth factor receptor (EGFR) family. Silencing ERBB2 down-regulates the production of ROS and up-regulates autophagy. Then increased autophagy can lead to autophagic cell death [93]. The results of various studies show autophagy under oxidative stress can be either protective or detrimental to cells. It is possible that the different effects of autophagy to RPE cells depend on degree and time of oxidative stress.

Degradation of photoreceptor outer segments is relied on RPE. In AMD, the function of RPE is impaired, causing the accumulation of a nondegradable and autofluorescent metabolite called lipofuscin in RPE [94]. A2E, the major component of lipofuscin, [95, 96] can aggravate oxidative damage to RPE cells. A2E probably activates autophagy through the Akt/mTOR signaling pathway [97, 98]. During this process, the Atg7-mediated pathway is not required [99]. Here, autophagy plays an important role in protecting RPE cells from the accumulation of toxic debris.

In general, in early-onset AMD, autophagy can compensate for disorders of RPE cells. In late AMD, reduced lysosomal enzymatic activity and autophagosomes inhibit autophagic clearance of lipofuscin, which means a decline of autophagy flux [100]. Gradually, the reduced autophagic clearance ability further increases the burden of inflammation and oxidative stress in RPE cells and accelerates the progression of AMD. The Prominin-1 (Prom1) gene can regulate autophagy by inhibiting mTOR in RPE. Induction of Prom1-dependent autophagy during aging may be an intrinsic defense mechanism, which enables the RPE cells to cope with increased oxidative burden [101].

*Diabetic Retinopathy (DR)*. More than 90% of patients with diabetes have type 2 diabetes mellitus with an onset age of over 55 years old. Most diabetic patients will develop some form of nonproliferative or proliferative complications of DR during diabetes, usually within 10–15 years. Increasing evidence suggests that retinal damage in diabetes patients is associated with the autophagic process [102]. Hyperglycemia triggers several metabolic signaling pathways that lead to the formation of advanced glycation end products (AGEs), oxidative stress, endoplasmic reticulum (ER) stress, and the secretion of proinflammatory cytokines. These changes lead to cell death by enhancing apoptosis, necroptosis and pathological autophagy [103]. These results suggest that the overexpression of autophagy may accelerate diabetic complications, such as DR. Synaptic contacts in the outer retina undergo increased autophagy in response to diabetes. Upregulation of autophagy occurs within the same time frame as outer retina damage and thus possibly represents the process leading to photoreceptor death in the early phase of DR [104]. High glucose can induce lysosomal impairment and inhibition of autophagy, which leads to a higher retinal Müller cell (rMC) apoptotic rate. Rapamycin can restore lysosomal activity, improving autophagy flux and relieving rMC apoptosis and VEGF production [105]. This indicates that improving lysosomal function is a possible therapeutic strategy for rMC survival. However, the effectiveness of this treatment needs more studies to support in vivo experiments in different contexts.

One of the pathological features of DR is microvascular leakage related to the destruction of pericytes. Extravasated modified heavily oxidized glycated LDL (HOG-LDL) also plays a critical role in DR [106–109]. Initial leakage may be mild and transient. When extravasated LDL accumulates, vicious cycles of damage may be established. Autophagy plays dual roles in human retinal capillary pericytes: it is protective under mild stress but promotes cell death under more severe stress (such as 200 mg/l HOG-LDL) [110]. Since autophagy has a dual influence on pericytes, autophagy-based therapies become confusing. Such therapies might be applicable only early in disease evolution when intraretinal stresses remain mild.

*Retinal Artery Occlusion (RAO)*Retinal artery occlusion (RAO) is one of the complications of cardiovascular diseases in the elderly [111]. There is a complex network between autophagy and cardiovascular disorders [112, 113]. Autophagy is directly or indirectly related to RAO. Various emboli can cause RAO, and the most common is carotid atherosclerotic plaque. In atherosclerosis, the accumulation of lipids and lipoproteins is an important pathophysiological change. These plaques are engulfed by macrophages to form foam cells, which contribute to the progression of atherosclerotic plaques [114]. Autophagy in atherosclerotic plaques can play either a beneficial or a detrimental role [112]. On the one hand, the accumulation of lipids can be cleared by selective autophagy (lipophagy) [115, 116]. Lipids are carried to lysosomes by autophagosomes, where they are degraded into free cholesterol and released from macrophages [117]. On the other hand, excessive autophagy in vascular smooth muscle cells can reduce collagen synthesis, which may destabilize plaque and provoke platelet aggregation and lesion thrombosis [112]. Previous studies indicate that autophagy is involved in the formation of atherosclerosis [118–121], but the accurate relationship between autophagy and atherosclerosis still needs further study. Therefore, the dysfunction of autophagy may contribute to the formation and abscission of atherosclerotic plaques, which finally leads to an increased risk of RAO.

The occurrence of RAO can result in focal retinal hypoxia-ischemia. In murine retinal microvascular endothelial cells (RMECs), autophagy is activated under hypoxia mainly through the AMPK/mTOR signaling pathway. However, the underlying mechanisms for autophagy changes and their effects on cell survival or death as well as retinal vascular diseases need further investigation [122].

### 3.4. Atophagy Pathway as Therapeutic Target in Ocular Diseases

As a potential target for novel therapeutics, autophagy has attracted considerable attention, especially in areas of neurodegeneration and cancer [123, 124]. Likewise, the link between autophagy and age-related eye diseases promote an intriguing question: whether the inhibition or promotion of autophagy could ameliorate diseases in certain conditions. Generally, autophagy can slow down or accelerate the development of ocular diseases in special situation, which make it difficult to explore the therapeutic targets of autophagy. Take example of AMD, rapamycin can inhibit mTOR to augment autophagy, which reveals a protective effect in RPE cells against the adverse effects of A2E [98]. Under oxidative stress, sodium tanshinone IIA sulfonate (STS) can decrease autophagy-related cell death by regulating signal pathway of autophagy in RPE cells. STS can activate the PI3K/AKT/mTOR pathway and restrict the initiation of autophagy. The expression of pivotal autophagic proteins, ATG3, ATG7, and ATG9 can be decreased by STS treatment, which inhibits the elongation of vacuoles and the formation of autophagosomes. In addition, Beclin 1, an autophagic protein, is important to nucleation of autophagy and also regulates apoptosis process. Its expression can be reduced by STS, which inhibits both autophagy and apoptosis in RPE cells [92]. In view of the results under different conditions, the mechanism of autophagy regulation in AMD at different stages deserves further investigation.

Despite the autophagy-targeted therapies have great potential, it still remains some obstacles. First, some drugs that can induce or inhibit autophagy present a low pharmacological specificity to target specific autophagy-related molecules or a special step of autophagy. STS mentioned above can intervene multiple molecules related to upstream and downstream autophagy signals. However, some autophagy-related molecules possess other functions besides participating in regulating autophagy pathway, which possibly brings some side effects [125]. For example, rapamycin plays a cytoprotective role through activating of autophagy by inhibiting mTOR in RPE cells and rMC [98, 105]. While the affected mTOR signaling pathway also influences protein synthesis, lipogenesis, and energy metabolism besides it functions as inhibiting autophagy [126]. Second, it is hard to monitor autophagic flux in vivo and lack reliable methods to discriminate between distinct forms of autophagy [127]. Therefore, how to directly evaluate the effect of drugs on autophagy in ocular tissues in vivo remains a problem to be resolved. Third, range of potentially useful doses of pharmacologic agents, reasonable course of treatment and optimal methods of drug deliver drugs are hard to be identified [125]. Fourth, deficiency in autophagy function may occur in the late stage of age-related eye diseases such as AMD, which make it weak effect to regulate autophagy pathway for slowing down the process of diseases. If therapeutic manipulation of autophagy either by genes or by drugs is applied to age-related eye diseases, molecular changes and exact roles of autophagy in age-related eye diseases must be clarified. At last, the bioavailability of autophagy-regulating drugs likely varies in different tissues [127]. Therefore, if this potential therapeutic method can be realized in age-related eye diseases, topical intraocular injection may obtain better curative effects and produce less side effects.

## 4. Summary and Conclusion

Autophagy is associated with a variety of age-related eye diseases. However, the specific relationship between autophagy and age-related diseases is still perplexing. Since autophagy is a complex regulatory process, the exact mechanism of autophagy remains unknown in some eye diseases. Generally, autophagy plays a dual role in eye diseases. In some cases, increased autophagy can reduce oxidative stress to maintain intracellular homeostasis, while in other conditions, the upregulation of autophagy results in autophagic cell death. Although many therapies based on autophagy have been proposed, external and animal experiments are not completely consistent with the human body, especially the condition of the elderly. All these factors lead to autophagy-based therapies that are more complex. In this field, we still have a long way to go.

## Data Availability

This article has no additional data.

## Supplementary Materials

Supplementary Materials Table 1: laboratory-based studies demonstrating the engagement of autophagy in age-related eye disease.
