# Supplementary material for: Autophagy and Age-Related Eye Diseases
Source: Biomed Res Int. 2019 Dec 14;2019:5763658. doi: 10.1155/2019/5763658 (PMC6948295; doi:10.1155/2019/5763658)
Supplement: Supplementary Materials — Table 1: laboratory-based studies demonstrating the engagement of autophagy in age-related eye disease. [file 5763658.f1.docx]

Table 1. Laboratory-based studies demonstrating the engagement of autophagy in age-related eye diseases

| Age-related eye diseases | Source | Year | Method | Results |
| --- | --- | --- | --- | --- |
| Dry eye disease | Zoukhri, D. et al.[1] | 2008 | In vivo | Lacrimal gland acinar cells are lost through apoptosis and autophagy is induced by inflammation. |
|  | Wang, N. et al.[2] | 2013 | In vitro | Lacritin can stimulate autophagy to restore homeostasis and plays a protective role in the dry eye. |
|  | Tony, L. et al.[3] | 2017 | In vitro | Impression cytology implicates a higher degree of autophagic cellular stress in ocular epithelial impression samples from patients with dry eye. |
| GCD2 | Choi, S. I. et al.[4] | 2012 | In vitro | Increased mutant-transforming growth factor β-induced protein (TGFBI) in autophagosomes indicates defective autophagy may play a critical role in the pathogenesis of GCD2. |
| FECD | Meng, H. et al.[5] | 2013 | In vivo/vitro | Dram1 is associated with increased autophagy. Overexpression of this gene in mouse and human FECD endothelial cells suggests a role for altered autophagy in FECD. |
|  | Kim, E. C. et al.[6] | 2013 | In vivo/vitro | Increased autophagy in lithium treated endothelium in a mouse model of FECD reveals autophagy may contribute to increased endothelial cell survival. |
|  | Benischke, A. S. et al.[7] | 2017 | In vitro | An elevation of LC3-II and LAMP1 and downregulation of Mfn2 in mitochondrial fractions are detected in human corneal endothelial cells. |
| Glaucoma | Porter, K. et al.[8] | 2014 | In vitro | Autophagy is activated in TM cells when subjected to biaxial static stretch (20% elongation), as well as in high-pressure perfused eyes (30 mm Hg). |
|  | Porter, K. et al.[9] | 2015 | In vitro | Dysregulated autophagic capacity was mTOR-dependent in cells isolated from the glaucomatous TM. |
|  | Sirohi, K. et al.[10] | 2015 | In vitro | Phosphorylation of Ser177 serves as a crucial role in M98K-OPTN-induced autophagosome formation, autophagy flux and retinal cell death. |

Table 1. Continued

| Age-related eye diseases | Source | Year | Method | Results |
| --- | --- | --- | --- | --- |
| Glaucoma | Shim, M. S. et al.[11] | 2016 | In vivo/vitro | E50K expression induces mitochondrial fission-mediated mitochondrial degradation and mitophagy in the axons of the glial lamina of aged E50K(-tg) mice in vivo. |
| Cataract | Costello, M. J. et al.[12] | 2013 | In vitro | Autophagy and mitophagy can be observed in epithelia from older human donor lenses. |
|  | Zhou, J. et al.[13] | 2016 | In vitro | Autophagic response of lens epithelial cells with increased LC3-II and p62 can be induced by oxidative stress in age-related cataract and Thioredoxin binding protein-2 (TBP-2) regulates this response. |
| AMD | Saadat, K. A. et al.[14] | 2014 | In vitro | Autophagy is augmented in the retinal pigment epithelial cell line ARPE-19 cultured in the presence of A2E and plays a cytoprotective role. |
|  | Zhang, J. et al.[15] | 2015 | In vitro | A2E induces autophagy and decreases cell viability in a concentration- and time-dependent manner. The augmentation of autophagy can reduce the adverse effects of A2E. |
|  | Perusek, L. et al.[16] | 2015 | In vivo/vitro | Atg7 deficiency doses not severely affect the health of RPE cells in mice and A2E accumulation is independent of Atg7-mediated autophagy in RPE cells. |
|  | Baek, A. et al.[17] | 2017 | In vitro | Upregulation of KRT8 (keratin 8) and its phosphorylation reduce the inhibition of autophagy which can protect RPE cells under oxidative stress. |
|  | Song, C. et al.[18] | 2017 | In vitro | P62is up-regulated in RPE cells under H2O2-induced oxidative stress and can promots autophagic activity. |
|  | Sheu, S. J. et al.[19] | 2019 | In vitro | Erb-b2 receptor tyrosine-protein kinase 2 (ERBB2) can modulate ATG4B for autophagy induction and increased autophagy leads to autophagic cell death in oxidative stress-stimulated ARPE-19 cells. |

Table 1. Continued

| Age-related eye diseases | Source | Year | Method | Results |
| --- | --- | --- | --- | --- |
| DR | Piano, I. et al.[20] | 2016 | In vivo | Initial dysregulation of autophagy results in photoreceptor death in the early phase of DR. |
|  | Jacqueline M. et al.[21] | 2016 | In vitro | High glucose upregulates autophagy but p62/SQTSM1 cargo can be accumulated due to lysosomal dysfunction, resulting in massive VEGF release and rMCs death. |
|  | Fu, D. et al.[22] | 2016 | In vitro | Autophagy increases in retina under diabetic models. It has a dual role in DR. |
| RAO | Ouimet, M. et al.[23] | 2012 | In vitro | Lipid loadingcan activate autophagy and cytoplasmic lipid droplets are delivered to lysosomes via autophagy. |
|  | Razani, B. et al.[24] | 2012 | In vivo | Dysfunction and deficiency of autophagy promotes atherosclerosis in part through inflammasome hyperactivation. |

*Abbreviations.* Type 2 granular corneal dystrophy (GCD2), Fuchs endothelial corneal dystrophy (FECD), Fuchs endothelial corneal dystrophy (FECD), trabecular meshwork (TM), Age-related macular degeneration (AMD), retinal pigment epithelial (RPE), diabetic retinopathy (DR), Retinal artery occlusion (RAO).

***References***

[1] D. Zoukhri, A. Fix, J. Alroy and C. L. Kublin, "Mechanisms of murine lacrimal gland repair after experimentally induced inflammation," *Invest Ophthalmol Vis Sci*, vol. 49, no. 10, pp. 4399-4406, 2008.

[2] N. Wang, K. Zimmerman, R. W. Raab, et al., "Lacritin rescues stressed epithelia via rapid forkhead box O3 (FOXO3)-associated autophagy that restores metabolism," *J Biol Chem*, vol. 288, no. 25, pp. 18146-18161, 2013.

[3] T. Lin, R. Filek, J. M. Wang, et al., "Impression cytology implicates cell autophagy in aqueous deficiency dry eye," *Clin Ophthalmol*, vol. 11, pp. 773-779, 2017.

[4] S. I. Choi, B. Y. Kim, S. Dadakhujaev, et al., "Impaired autophagy and delayed autophagic clearance of transforming growth factor beta-induced protein (TGFBI) in granular corneal dystrophy type 2," *Autophagy*, vol. 8, no. 12, pp. 1782-1797, 2012.

[5] H. Meng, M. Matthaei, N. Ramanan, et al., "L450W and Q455K Col8a2 knock-in mouse models of Fuchs endothelial corneal dystrophy show distinct phenotypes and evidence for altered autophagy," *Invest Ophthalmol Vis Sci*, vol. 54, no. 3, pp. 1887-1897, 2013.

[6] E. C. Kim, H. Meng and A. S. Jun, "Lithium treatment increases endothelial cell survival and autophagy in a mouse model of Fuchs endothelial corneal dystrophy," *Br J Ophthalmol*, vol. 97, no. 8, pp. 1068-1073, 2013.

[7] A. S. Benischke, S. Vasanth, T. Miyai, et al., "Activation of mitophagy leads to decline in Mfn2 and loss of mitochondrial mass in Fuchs endothelial corneal dystrophy," *Sci Rep*, vol. 7, no. 1, pp. 6656, 2017.

[8] K. M. Porter, N. Jeyabalan and P. B. Liton, "MTOR-independent induction of autophagy in trabecular meshwork cells subjected to biaxial stretch," *Biochim Biophys Acta*, vol. 1843, no. 6, pp. 1054-1062, 2014.

[9] K. Porter, J. Hirt, W. D. Stamer and P. B. Liton, "Autophagic dysregulation in glaucomatous trabecular meshwork cells," *Biochim Biophys Acta*, vol. 1852, no. 3, pp. 379-385, 2015.

[10] K. Sirohi, A. Kumari, V. Radha and G. Swarup, "A Glaucoma-Associated Variant of Optineurin, M98K, Activates Tbk1 to Enhance Autophagosome Formation and Retinal Cell Death Dependent on Ser177 Phosphorylation of Optineurin," *PLoS One*, vol. 10, no. 9, pp. e0138289, 2015.

[11] M. S. Shim, Y. Takihara, K. Y. Kim, et al., "Mitochondrial pathogenic mechanism and degradation in optineurin E50K mutation-mediated retinal ganglion cell degeneration," *Sci Rep*, vol. 6, pp. 33830, 2016.

[12] M. J. Costello, L. A. Brennan, S. Basu, et al., "Autophagy and mitophagy participate in ocular lens organelle degradation," *Exp Eye Res*, vol. 116, pp. 141-150, 2013.

[13] J. Zhou, K. Yao, Y. Zhang, et al., "Thioredoxin Binding Protein-2 Regulates Autophagy of Human Lens Epithelial Cells under Oxidative Stress via Inhibition of Akt Phosphorylation," vol. 2016, pp. 4856431, 2016.

[14] K. A. Saadat, Y. Murakami, X. Tan, et al., "Inhibition of autophagy induces retinal pigment epithelial cell damage by the lipofuscin fluorophore A2E," *FEBS Open Bio*, vol. 4, pp. 1007-1014, 2014.

[15] J. Zhang, Y. Bai, L. Huang, et al., "Protective effect of autophagy on human retinal pigment epithelial cells against lipofuscin fluorophore A2E: implications for age-related macular degeneration," *Cell Death Dis*, vol. 6, pp. e1972, 2015.

[16] L. Perusek, B. Sahu, T. Parmar, et al., "Di-retinoid-pyridinium-ethanolamine (A2E) Accumulation and the Maintenance of the Visual Cycle Are Independent of Atg7-mediated Autophagy in the Retinal Pigmented Epithelium," *J Biol Chem*, vol. 290, no. 48, pp. 29035-29044, 2015.

[17] A. Baek, S. Yoon, J. Kim, et al., "Autophagy and KRT8/keratin 8 protect degeneration of retinal pigment epithelium under oxidative stress," *Autophagy*, vol. 13, no. 2, pp. 248-263, 2017.

[18] C. Song, S. K. Mitter, X. Qi, et al., "Oxidative stress-mediated NFkappaB phosphorylation upregulates p62/SQSTM1 and promotes retinal pigmented epithelial cell survival through increased autophagy," *PLoS One*, vol. 12, no. 2, pp. e0171940, 2017.

[19] S. J. Sheu, J. L. Chen, Y. S. Bee, S. H. Lin and C. W. Shu, "ERBB2-modulated ATG4B and autophagic cell death in human ARPE19 during oxidative stress," *PLoS One*, vol. 14, no. 3, pp. e0213932, 2019.

[20] I. Piano, E. Novelli, L. Della Santina, et al., "Involvement of Autophagic Pathway in the Progression of Retinal Degeneration in a Mouse Model of Diabetes," *Front Cell Neurosci*, vol. 10, pp. 42, 2016.

[21] Jacqueline M.Lopes de Faria, Diego A. Duarte, Chiara Montemurro and A. Papadimitriou, "Defective Autophagy in Diabetic Retinopathy," 2016.

[22] D. Fu, J. Y. Yu, S. Yang, et al., "Survival or death: a dual role for autophagy in stress-induced pericyte loss in diabetic retinopathy," *Diabetologia*, vol. 59, no. 10, pp. 2251-2261, 2016.

[23] M. Ouimet and Y. L. Marcel, "Regulation of lipid droplet cholesterol efflux from macrophage foam cells," *Arterioscler Thromb Vasc Biol*, vol. 32, no. 3, pp. 575-581, 2012.

[24] B. Razani, C. Feng, T. Coleman, et al., "Autophagy links inflammasomes to atherosclerotic progression," *Cell Metab*, vol. 15, no. 4, pp. 534-544, 2012.
